# Ultrathin Ti_3_C_2_T_x_ MXene sheets with high electrochemically active area anchored Pt boosting hydrogen evolution

**DOI:** 10.1016/j.heliyon.2023.e19197

**Published:** 2023-08-19

**Authors:** Zicong Yang, Yanhui Chen, Ge Chen, Jinshu Wang, Hongyi Li

**Affiliations:** a Faculty of Materials and Manufacturing, Beijing University of Technology, 100124, Beijing, China; b Beijing Key Laboratory for Green Catalysis and Separation, Faculty of Environment and Life, Beijing University of Technology, Beijing, 100124, China

**Keywords:** Hydrogen evolution reaction, Ultrathin MXene, Electrochemically active area, Little restacking

## Abstract

To reduce platinum usage, ultrathin MXene sheets with little restacking effect were prepared. The ultrathin MXene was prepared by a two-step etching process, which showed high specific surface area with low charge transfer resistance. The sample showed a double layer capacity of 64.98 mF cm^−2^, which is 14 times as large as that of ordinary HF prepared MXene, indicating a larger electrochemically active surface area. It showed a much better HER performance of ∼190 mV at 10 mA cm^−2^. The better performance attributes to 0.4 wt% Pt loaded. The Pt loaded MXene exhibited a better HER performance of ∼75 mV at 10 mA cm^−2^ and a Tafel slope of 61.7 mV·dec^−1^ close to 40 wt% commercial Pt/C. The sample performed better than Pt/C in a 3 h chronopotentiometry test and hardly changed in ECSA after the cyclic experiment. With more Pt loading, the sample delivered better HER performance than Pt/C in the LSV test (∼51 mV at 10 mA cm^−2^). This work provides an effective route for the preparation of ultrathin MXene sheets with larger electrochemically active area and more active sites for Pt loading, leading to superior HER performance.

## 1 Introduction

Carbon neutralization becomes a hotspot in today's energy area. Hydrogen energy is clean and efficient, which makes it a more ideal energy source compared with traditional fossil fuel [1,2]. Electrocatalyst hydrogen evolution reaction (HER) is the highly active measure to produce hydrogen by the splitting of water [3]. Noble metals including platinum (Pt) or ruthenium (Ru) are the most promising electrocatalyst for HER [[4], [5], [6]]. Yet the high-cost and low reserves limit its possibility of wide range practical application [7]. Therefore, two major solution is considered as appropriate measurements. One is finding certain replacement of noble metal component. There are lots of efforts being made to find non-noble metal materials including alloys, transition metal compounds and carbonaceous nanomaterials [[8], [9], [10]]. Nevertheless, these materials still need further modification for practical use such as doping [11], controlling the crystallinity and increasing the active size by changing the morphology [12,13]. Another measurement is making usage of Pt element in a most efficient way. One of the most common strategies is taking the carbon-based materials as substrates [14]. Noble metal particle or atoms anchor to the surface exposed to achieve good HER performance with little noble materials applied [[15], [16], [17]].

MXene, a new kind of transition metal carbides firstly found in 2011, owning its unique 2D layered structure made by carbon atomic layer sand transition metal atomic layers has shown its application in many fields like batteries and supercapacitors [18,19]. Due to its 2D structure, it owns many active sites for HER [20]. Furthermore, the transition metal atoms and carbon atoms inside give MXene superior metallic conductivity, high reducibility and hydrophilic properties [21]. Hence, sufficient researches are made to explore its possibility in HER [22]. The researches mainly focus on adjusting the abundant function group on the surface [23], producing vacancies or compounding with other materials [24,25]. All the experimental schemes emphasize the microstructure of MXene or the composite made of MXene. There are few works focusing on the morphology of MXene itself. Shen et al. used Ti_3_C_2_T_x_ MXene combined with nitrogen-doped graphene to make a porous composite structure [26]. The composite exhibits a low onset potential and a relatively low Tafel slope compared with pure Ti_3_C_2_T_x_ MXene. However, the nonuniformly morphology and restacking effect of the 2D MXene sheets still hinder its activity in catalyst and further restrict its practical use in HER [27]. Seh et al. proved that HER performance of Mo_2_CT_x_ is improved when Mo_2_CT_x_ is in delaminated form (d-Mo_2_CT_x_) [28]. So the study focused on morphology design of MXene seems urgent to solve the restacking problem to make the 2D MXene sheets fully exposed in electrolyte so that more active spots will be involved in HER process.

In this work, a two-step etching procedure is used to synthesize ultrathin Ti_3_C_2_T_x_ MXene nanosheets with little restacking happens. These ultrathin sheets exhibit high specific surface area indicating abundance active sites. Compared with addition of dimethyl sulfoxide, other organic amines or using chemical vapor deposition to keep the delamination of layers [[29], [30], [31]], our strategy is simple, environmental-friendly and easy to operate. Besides, the charge transfer resistance evidently decreases and the double-layer capacitance highly improved compared to stacked 2D MXene. These advantages lead to a better HER performance including lower onset overpotential and lower Tafel slope comparing with traditional 2D MXene. The ultrathin MXene performed well in HER performance with loading Pt particles as well. Most importantly, since there are rich species of MXene family besides Ti_3_C_2_T_x_ MXene [32]. Our procedure can further be applied to other MXene. This work presents a brand-new idea in preparation of ultrathin MXene sheets with little restacking so that MXene with excellent HER performance can be obtained.

## 2 Experimental

### 2.1 Chemicals and materials

All chemicals were used as received without further purification. Chloroplatinic acid (H2PtCl6·6H2O, 99.9%) was purchased from Mreda. Ti_3_AlC_2_ MAX precursor was purchased from Kaixi Ceramic Materials Co., Ltd. Lithium fluoride (LiF), Potassium hydroxide (KOH) and hydrochloric acid (HCl) were purchased from Macklin.

### 2.2 Synthesis of ultrathin MXene and two-dimensional MXene

The ultrathin MXene was prepared by 2-step etching procedure. 2 g Ti_3_AlC_2_ MAX (500 mesh) was added slowly into 20 mL of 6 M KOH for etching in case the Al(OH)_3_ covering MXene hindering the following chemical reaction between the MXene and alkaline. After magnetic stirring for 72 h at room temperature. The powder was washed by DI water and collected by centrifugation at 6000 rpm for 3 min for several times until pH approaching ∼7. The product was vacuum dried at 40 °C for 24 h and 72 h MAX was made. 1 g LiF and 20 mL of 12 M HCl was mixed to produce HF. 1 g of 72 h MAX was slowly added in the solution above and magnetic stirring at 35 °C for 24 h The suspension was washed then centrifuged at 7000 rpm for 5 min until the pH of the supernatant is close to 6. The sediment was collected and ultrasonically treated under Ar atmosphere in DI water under ice-bath for 30 min The above suspension was centrifuged under 3000 rpm for 40min. The final product was black suspension, which was kept in fridge overnight and vacuum dried by lyophilization. The ultrathin MXene was successfully obtained noted as UT.

The MXene was prepared with no KOH etching envolved by simply adding 1 g MAX in 1 g LiF and 20 mL 12 M HCl instead of 72 h MAX. Following procedure stay the same. The final product was noted as 2D.

### 2.3 Synthesis of Pt/UT MXene and Pt/2D MXene

50 mg of UT were added into 50 mL DI water and sonicated under ice-bath for 5 min to make homogeneous solution. Proper amount of H_2_PtCl_6_·6H_2_O (25 g/100 mL) was added according to the proportion. Then the resultant was kept under continuous stirring for 10 min and sonicated for 30 min under ice-bath to make sure the H_2_PtCl_6_·6H_2_O was evenly distributed in the suspension. The suspension above was kept in fridge overnight and freeze-dried. Based on the weight of Pt contained in the sample verified by ICP-OES results. (e.g. Pt1/UT means the weight ratio of Pt in this sample is 1% of the one in 40 wt% Pt/C, which is 4 μg Pt in 1 mg catalyst), Pt1/UT, Pt10/UT (40 μg in 1 mg catalyst) and Pt40/UT (240 μg in 1 mg catalyst) were obtained.

For the Pt/2D, the procedure was the same except for the 2D MXene was added instead of UT. Eventually, Pt1/2D was made.

### 2.4 Characterization

The morphology and chemical composition were observed under scanning electron microscope (SEM) (SU8020, Hitachi), energy dispersive X-ray spectroscopy (EDS) mapping, transmission electron microscope (TEM) (Talos F200X-G2, FEI) and ICP-OES (OPTIMA7000DV, AGILENT). N_2_ adsorption-desorption isotherm was obtained using Brunauer-Emmett-Teller (BET) method by ASAP2460. X-ray diffraction (XRD) patterns were obtained from Shimadzu XRD-7000 using Cu Kα radiation (λ = 0.154 nm). X-ray photoelectron spectroscopy (XPS) analysis was performed by ESCALAB 250Xi spectrometer. Atomic force microscope (AFM) are performed on Bruker Multimode 8.

### 2.5 Electrochemical measurement

Electrochemical measurement was performed on an AMETEK PARSTAT MC. A three-electrode system was made using the carbon paper (1cm × 1 cm) coated with electrocatalyst as working electrode, saturated calomel and a graphite rod as reference and counter electrode, respectively. For assembly of working electrode, 5 mg electrocatalyst were added in a solution consist of 650 μL DI water and 300 μL ethanol and 50 μL 5% Nafion. The solution above was sonicated for 30min for complete mixing. The solution was drop cast on the carbon paper at the amount of 200 μL total to make sure the electrolyte mass loading was 1 mg cm^−2^.

Then the carbon paper was clamped on a platinum electrode clip. All the measurements were performed in 0.5 M H_2_SO_4_ and all potentials were referenced to the reversible hydrogen electrode (RHE) according to equation [E_(RHE)_ = E_(SCE)_+0.059 pH + 0.241 V]. To obtain the double layer capacitance (C_dl_), cyclic voltammetry (CV) tests were carried out from 20 mVs^−1^ to 100 mVs^−1^. To investigate the stability, CV was performed at 100 mVs^−1^ scan rate. The chronopotentiometric was performed at −10 mA. Electrochemical impedance analysis (EIS) was taken at a frequency ranging from 0.01 Hz to 100 k Hz. 40 wt% commercial Pt/C (Pt/C) was used for comparison.

## 3 Result and discussion

### 3.1 Morphology and structural analysis

**Scheme 1:**
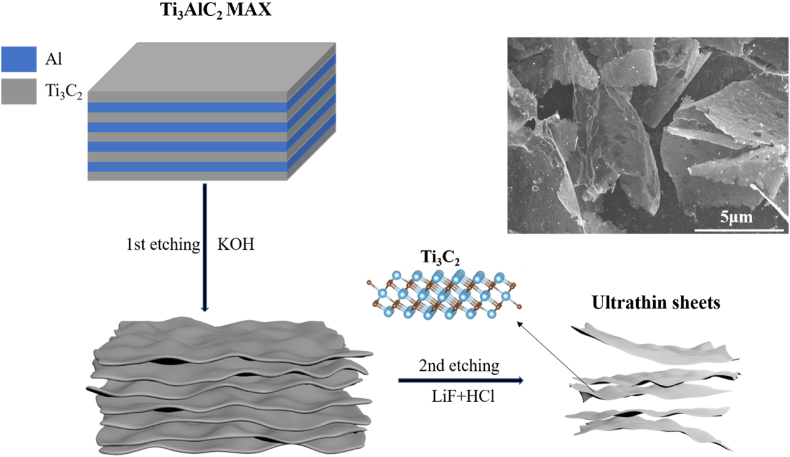
Schematic diagram for the synthesis of UT.

The synthesis route is shown in Scheme 1. The precursor MAX presents a bulk morphology according to the SEM images in Fig. S1a, b. The bulk is made of multiple layers of Al and Ti_3_C_2_ as is shown in the diagram. The Al^3+^ concentration in the alkaline after 72 h treatment is 43.1 mg L^−1^. The 20 mL alkaline was diluted to 2 L for ICP test. There are 86 mg Al^3+^ in the resultant alkaline, which means during the first-time etching process, the KOH removed 30% of the Al content in the bulk. Fig. S1c, d show the morphology of the MAX precursor after 72 h alkaline treatment. The MAX transformed into thick flake due to the KOH etching. Since the incomplete removement of Al, there are still some connections remaining. After the second etching process with HF in-situ synthesized by LiF and HCl, the Al layer is completely removed. We also performed 96 h of alkaline treatment and the corresponding morphology can be observed in Fig. S1e, f. The morphology of MXene is massively changed. Nanowire-like morphology is shown. But the oxidization of Ti in Ti_3_C_2_T_x_ happened and TiO_2_ was formed. This undoubtedly hinder the HER performance. In this regard, 96 h is too long for alkaline treatment. Compared with direct use of HF, the in-situ generated HF by LiF and HCl brings less fluorine on the basal plane of MXene sheets [33]. Furthermore, with abundant –OH, –O introduced by KOH in the first step, the oxygen containing group occupies most of the etched surface of MXene sheets. With comprehensive effect of these two factors, the –F brought by HF etching will be limited [34]. Since that the presence of fluorine hinders HER performance, this ultrathin MXene sheets synthesized by two-step etching procedure possess better HER activity than those made by simply one-step etching by HF.

**Fig. 1:**
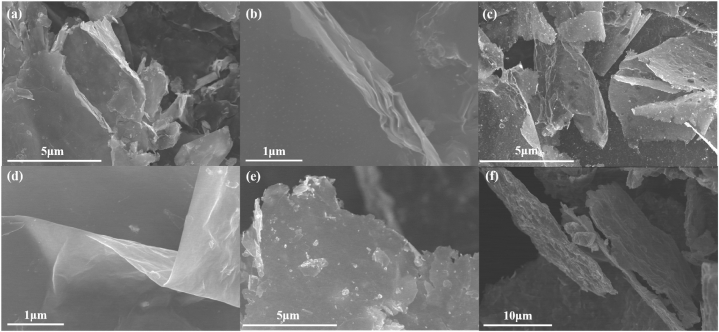
Morphology of different catalysts synthesized: (a–b) SEM image of 2D MXene, (*c*–d) Ultrathin MXene. (e) Pt1/UT, (f) Pt10/UT.

The morphology of UT and 2D can be observed in Fig. 1. With two-step etching applied, the effect on the thickness of MXene sheets can be evidently discovered. From Fig. 1a, the stacked MXene shows in a rather large part. A closer look of stacked MXene sheets is shown in Fig. 1b. The stacked structure with a thickness of ∼500 nm can be clearly observed. As for the UT morphology, a crumpled ultrathin sheet is shown in Fig. 1c. From a deeper look at the wrinkle part in Fig. 1d, the thickness can be roughly evaluated. The accurate thickness of UT and 2D are shown in Fig. S2 evaluated by AFM. Fig. S2a shows the thickness of UT is 2.0 nm ± 0.3 nm while the 2D exhibits a much larger thickness of 10.5 nm ± 0.5 nm as shown in Fig. S2b. As With the successful preparation of ultrathin MXene, the excellent exfoliation of MXene sheets created by two-step etching possess bigger specific surface area from the BET result in Fig. S3. The UT exhibits larger specific surface area of 12.07 m^2^ g^−1^ while 2D only shows 2.535 m^2^ g^−1^. The nearly five times larger value means the UT can provide more active sites for HE R. Fig. S4 shows three other areas of UT proving the ultrathin sheets existed in a large scale other than one specific area. Observed from a larger field of vision, the well exfoliated ultrathin sheets occupy the major part representing the homogeneity of UT.

Apart from larger specific surface area, the element compositions of UT and 2D were evaluated in EDS mapping in Fig. S5. The mapping shows Ti and F in certain areas selected from both 2D and UT, suggesting the UT got less F element compared with 2D. Less F left means less –F group brought by the HF. The –F group attenuates the activity of HER [35,36], so the UT MXene is more suitable for HER than 2D.

To further evaluate the ability of UT to load Pt element. H_2_PtCl_6_·6H2O was used as Pt source. The Pt^4+^ in H_2_PtCl_6_·6H2O would be reduced to Pt^2+^ and Pt^0^ (Pt particle) on the surface of MXene sheets. Fig. 1e Shows the morphology of Pt1/UT. With little Pt element introduced from H_2_PtCl_6_·6H2O in UT, the ultrathin sheets maintained meanwhile the Pt particles are hardly found on the surfaces of UT sheets. To acknowledge the specific combination mode of Pt element and UT, more Pt element was introduced in UT. Therefore, 10 times the amount of Pt element were added in the sample of UT. The morphology of Pt10/UT are apparently changed in comparison with Pt1/UT. The surfaces turned rough and thick because of the Pt particles generated (Fig. 1f). The elemental mapping of Pt in Pt10/UT in Fig. S6 gives a clear evidence of Pt particles uniformly generated on the surface and were distributed on the surface of UT sheets in a relatively large area. Although they become thick because of the growing Pt particle，the sheets still keep in uniform shape. Moreover, fully exposed Pt particles can maximize its effect in HER reaction.

**Fig. 2:**
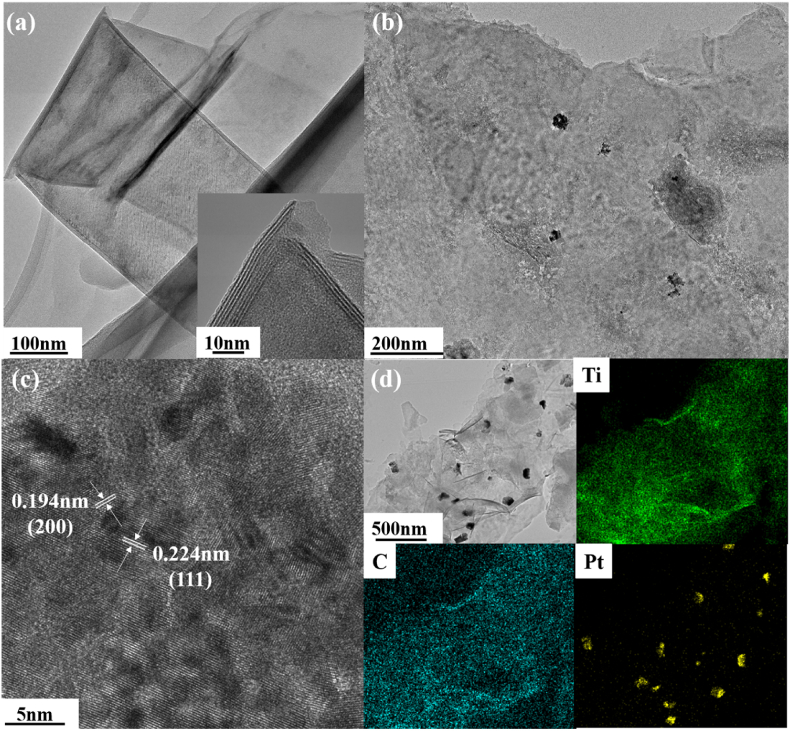
TEM image of: (a) Ultrathin MXene (inset: selective area at different magnification), (b–c) Pt1/UT, (d) TEM elemental mapping Ti, C, Pt in Pt1/UT.

TEM image is shown in Fig. 2a where a single sheet consists of folded layers of UT is shown. It can be inferred that the thickness of UT is relatively small. Same conclusion can be proved by the observation of the edge in the inset of Fig. 2a, the thickness is less than 10 nm with 4 layers stacked together. The thickness of single layer is extremely close to the single layer of Ti_3_C_2_ according to previous report [37]. The single sheet morphology can be observed in other areas investigated by TEM shown in Fig. S7. Fig. 2b shows the clearly morphology of Pt1/UT. The Pt particles were embedded on the MXene sheet. Besides, the size of Pt particles is below 100 nm, which means the Pt element shows as nanoparticles. Fig. 2c gives the closer look of the crystal lattice of Pt nanoparticles. The (200) and (111) planes of Pt with interplanar spacing of 0.194 nm and 0.224 nm are marked respectively. This shows the great crystallinity of Pt nanoparticles in Pt1/UT. Fig. 2d exhibits the mapping of Pt, Ti and C in selected area of Pt1/UT. Ti and C distributed widely in this area means the existence of UT. With bigger area under investigation, the uniformly distributed Pt nanoparticles maintain in nanoparticle form. The average size of the particle in this figure is 83 nm with a standard error of 18.6 nm. The Pt element is successfully introduced in UT. This is proved by the TEM observation.

Interestingly, from observation of photograph of different inks shown in Fig. S8, the 2D, UT and Pt1/UT maintain the form of suspension after 48 h of standing while the other samples turn into precipitation. This phenomenon indicates UT having great structure maintenance after a small amount of Pt loaded. On the other hand, Pt1/2D does not maintain the same form. This means the multilayer structure of 2D hinders its capability of loading Pt. With more Pt addition, the Pt10/UT and Pt40/UT turned into precipitation as well. More Pt introduced caused the restacking effect in both UT and 2D, which leads to the formation of precipitation.

**Fig. 3:**
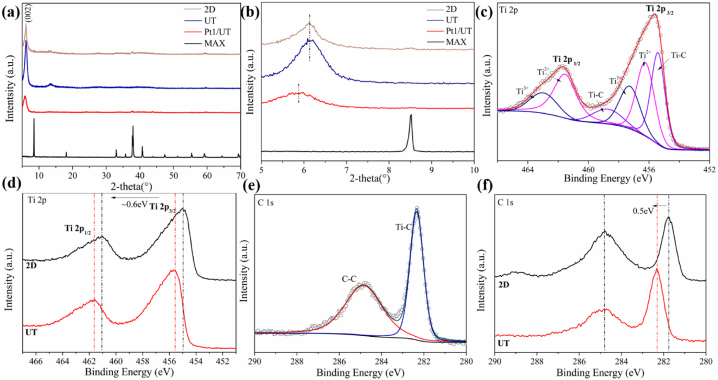
(a) XRD patterns of 2D, UT, Pt1/UT, MAX within the 2-thera range of 5°-70° and (b) 5°-10°. (c) XPS spectra of Ti 2p of UT, (d) Ti 2p of 2D and UT. (e) C 1s of UT, (f) C 1s of 2D and UT.

The XRD results are displayed in Fig. 3a. The patterns of 2D and UT are compared with the one of MAX precursor, the diffraction peak at ∼8.5° representing (002) of Ti_3_AlC_2_ shifts to a lower angle (∼6.2°) while others disappear after etching performed. This means the Al element in Ti_3_AlC_2_ was successfully removed while the interlayer spacing of (002) increased because of the introduction of hydroxyl and fluorine groups [[38], [39], [40]]. The increase of interlayer is beneficial to ion and electron transmission between Ti_3_C_2_T_x_ nanosheets. The 5°-10° part of the XRD patterns are exhibited in Fig. 3b. With the comparison of XRD patterns of UT and 2D, the (002) diffraction peak of UT is sharper, which indicates better crystallinity of UT than 2D. Excellent crystallinity indicating the regular structure of UT and the pure crystal phase, makes electron and ion transfusion easier and more efficient. Since Pt element introduced, the (002) diffraction peak shifts to a lower angle (∼5.8°). The interlayer spacing further expands and the intensity decreases because of loaded Platinum. However, because the amount of Pt loaded in Pt1/UT was far too little to be detected by XRD [41,42]. No evident diffraction peak of Pt is shown.

XPS spectra of different sample was taken. Ti 2p spectra of UT is shown in Fig. 3c. The double peaks of Ti 2p_1/2_ and Ti 2p_3/2_ show 3 doublets represent 3 kinds of bonds marked as Ti^3+^, Ti^2+^ and Ti–C. The Ti^3+^ stands for C–Ti–O and Ti^2+^ for C–Ti–OH [27,43]. Ti–C and Ti^3+^ occupy the largest portion. Same result can be concluded from Ti 2p spectra of 2D in Fig. S9a. The peaks of Ti 2p spectra are compared in Fig. 3d. Although the surface group and chemical state of Ti element are familiar between 2D and UT, the peaks of UT shift to a higher position. The ∼0.6 eV higher binding energy of UT means tighter bonds are generated in UT. C 1s spectra is shown in Fig. 3e. The bonding type of C in UT only concludes C–C bond located at 284.86 eV and Ti–C bond at 282.35 eV. This means the C element remain its structure between two Ti atomic layers in Ti_3_C_2_T_x_ MXene. From observation of C 1s in 2D (Fig. S9b), the chemical state of C and related bonding type are more complicate compared with UT. Apart from the Ti–C and C–C bond, the two C–O peaks are shown at 286.51 eV and 288.97 eV respectively [23]. This means part of the C atoms in 2D bonding with O atoms. The spectra show a gap between 2D and theoretical model Ti_3_C_2_T_x_ which is sandwich-like, consisting of two C atomic layers in between three Ti atomic layers. The C 1s spectra of 2D and UT are shown in Fig. 3f. With C–C peak stays in the same location, Ti–C peak of UT shifts to a higher bonding energy. This indicates the Ti–C bond in UT is stronger than 2D. Shorter bonding means smaller thickness and the closer contact between carbon layers and titanium layers keeps UT in good shape and stabilize the stabilize the structure in HER progress.

**Fig. 4:**
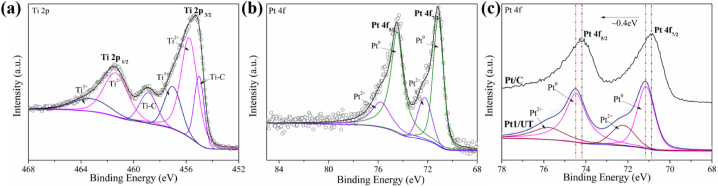
(a) XPS spectra of Ti 2p of Pt1/UT, (b) Pt 4f of Pt1/UT, (c) Pt 4f of Pt/C and Pt1/UT.

The survey spectrums of UT and Pt1/UT are shown in Fig. S10 With Pt 4f peaks observed at ∼75 eV, Pt element was successfully introduced in UT [42]. Fig. 4a shows the Ti 2p of Pt1/UT, the chemical state of Ti turns into Ti^2+^ doublet as the major portion instead of Ti–C shown in UT [43]. The change of peaks related to the change of valence of Ti element. The valence of Ti increases after the addition of Pt element in UT. The Ti 2p spectra of Pt1/UT and UT are shown inFig. S11. Except the change of valence state, the peak of Ti 2p_1/2_ and Ti 2p_3/2_ shift to a lower location after Pt additive. This phenomenon may be caused by the formation of Pt–O bond that weakens the strength of Ti bond. Therefore, Pt 4f spectra of Pt1/UT is shown in Fig. 4b. The Pt 4f_5/2_ and Pt 4f_7/2_ consist of 2 component doublets as Pt^2+^ at ∼75.81, ∼72.24 eV and Pt^0^ at ∼74.46, ∼71.16 eV with a ratio of 1:3 [44]. This shows the presence of Pt element changes from Pt^4+^ in H_2_PtCl_6_ as initial state to Pt^2+^ assigned as Pt–O bond represent Pt (OH)_2_ or PtO and Pt^0^ as metallic Pt in Pt1/UT [45]. This indicates the Pt^4+^ was successfully reduced to Pt^2+^ and Pt^0^. And correspondingly, the Ti element was oxidized to a higher valence state, which is consistent with the Ti 2p spectra. Pt 4f spectra of Pt1/UT is also compared with the one of Pt/C in Fig. 4c as is shown, the chemical states of these two samples are close. Furthermore, the higher peak position of Pt 4f_5/2_ and Pt 4f_7/2_. This indicates the Pt1/UT owns stronger interaction of Pt and Pt–O than Pt/C [41].

### 3.2 Electrochemical HER measurement

**Fig. 5:**
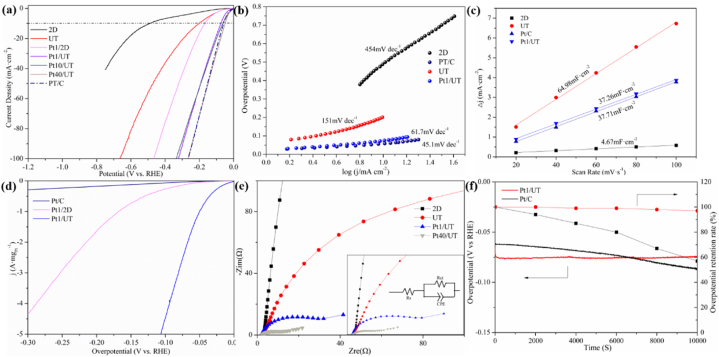
The electrocatalysis performance of synthesized catalyst in 0.5 M H_2_SO_4_. (a) Linear scan voltammetry curves of 2D, UT, Pt1/2D, Pt1/UT, Pt10/UT, Pt40/UT, Pt/C. (b) Tafel slope of 2D, Pt/C, UT, Pt1/UT. (c) Double-layer capacitance (C_dl_) plots of 2D, UT, Pt/C, Pt1/UT. (d) Mass activity of Pt/C, Pt1/2D, Pt1/UT. (e) EIS spectras of 2D, UT, Pt1/UT, Pt40/UT with equivalent circuit shown. (f) 3 h Chronopotentiometry test and overpotential retention rate of Pt1/UT and Pt/C.

The HER performances are investigated. The linear scan voltammetry (LSV) is shown in Fig. 5a. The UT shows a much better performance than 2D. Limited by severely stacked structure and rich –F on the surface [33,34], 2D exhibit poor overpotential (∼491 mV vs. RHE) at 10 mA cm^−2^. However, the UT shows excellent performance at the same current density due to the structural advantage (∼190 mV vs. RHE). Then the Pt loading samples are investigated. Undoubtedly, with the addition of Pt element, the HER performance evidently increases [46]. Although the overpotential of Pt1/2D (∼166 mV vs. RHE) is much smaller than previous 2D, the Pt1/UT shows an excellent HER performance of ∼75 mV at 10 mA cm^−2^. Larger specific area created by two-step etching process gives UT more edge and surface. These newly found morphology not only provides more active sites for HER but also improves the application of Pt nanoparticles effectively. Then the proportion of Pt in UT was adjusted. Pt10/UT which is covered with plenty of Pt nanoparticles observed in SEM further improved the HER performance (∼63 mV vs. RHE at 10 mA cm^−2^). And when the Pt content reaches 40% the Pt content of Pt/C, the overpotential of 40Pt/UT (∼51 mV vs. RHE) is better than the one of Pt/C (57 mV vs. RHE) at 10 mA cm^−2^. But the Pt loading of Pt40/UT is far beyond the upper limit of UT can handle so the Pt nanoparticles began to peel off and dispersed in electrolyte after LSV test, which makes the further investigation of Pt40/UT unnecessary. The column pattern i n Fig. S12 shows visually about the comparison between the samples in this work and other previously reported noble metal-based or 2D material-based catalysts in HER. The Pt40/UT shows best HER performance while PT10/UT and PT1/UT still remains competitive among these catalysts. The UT even perform superior against some noble-metal catalysts.

Tafel slopes are compared in Fig. 5b to take a deeper observation of HER kinetics. The UT exhibits a much better performance for HER than 2D (454 mV·dec^−1^) with a Tafel slope of 151 mV·dec^−1^. This means the UT itself is suitable for HER. With further loaded with little Pt element, Pt1/UT shows an excellent kinetic by a value of 61.7 mV·dec^−1^, which is close to the value of Pt/C (45.1 mV·dec^−1^). Based on the value of slope and compared with Volmer reaction (H_3_O^+^+e^−^ + M→H_ad_ + H_2_O) at 120 mV·dec^−1^ and Heyrovsky reaction (MH_ad_ + e^−^ + H_3_O^+^→M + H_2_+H_2_O) at 40 mV·dec^−1^ [47], It can be inferred that the Pt1/UT as well as UT show a Volmer-Heyrovsky route in the HER experiment while 2D shows Volmer reaction as the rate-determined step. This also means that with the introduction of Pt in UT, the Volmer reaction accelerated and the rate change limiting step of HER kinetics changed from first step (H^+^→H_ad_) to second (2H_ad_→H_2_) [48,49].

The electrochemically active surface area (ECSA) of the electrocatalysts are evaluated by the electrochemical C_dl_ [50,51]. To obtain the C_dl_, cyclic voltammetry (CV) was taken at multiple scan rates in a non-faradic area from 0.35 to 0.55 V vs. RHE. The CV curves of 2D, UT, Pt/C and Pt1/UT are displayed in Fig. S13a-d. The current density values at 0.441 V vs. RHE were taken. Fig. 5c shows the C_dl_ of three electrocatalysts. 2D has the lowest C_dl_ at 32.3 mF cm^−2^, which indicate the lowest ECSA. It is worth noting that Pt/C shows a relatively lower C_dl_ (37.7 mF cm^−2^) than UT (65.5 mF cm^−2^) and still exhibit great HER performance. For the UT, it owns largest C_dl_ value indicating biggest ECSA. This gives UT the reason of possessing better HER performance than 2D since the composition of these two samples is consistent. With no Pt involved in UT, its large ECSA means superior HER performance. After being connected with Pt element, the C_dl_ of UT decreases to 37.2 mF cm^−2^. Despite this means the ECSA is influenced by the Pt nanoparticles to a rather low level, it is still close to the level of Pt/C from investigation. The addition of little Pt is beneficial to HER, so the Pt1/UT still deliver much better performance than UT.

The current densities of Pt1/2D, Pt1/UT and Pt/C are normalized to the loading of Pt to investigate the mass activity of catalysts [43,52]. The Pt/C has the best performance in LSV test but since the 40 wt% loading of Pt in commercial Pt/C catalyst, the mass activity is relatively low in Fig. 5d. The layered structure gives both 2D and UT better efficiency in utilization of Pt. The mass activity is much higher than Pt/C. But the UT obviously exhibit more outstanding mass activity. This benefit from UT possessing less stacked layer structure than 2D brought by two-step etching process. Better structure brings the mass activity of Pt1/UT (∼4522 mA·mg_pt_^−1^) ∼17 times better than Pt1/2D (∼269 mA·mg_pt_^−1^) at 0.1 V vs. RHE. This means the Pt particles anchored on UT are more capable of delivering great HER performance.

EIS is adopted to reveal the HER kinetics behavior. The arches represent the charge transfer resistance (R_ct_). R_ct_ was evaluated by the equivalent circuit in Fig. 5e [53]. The UT shows a smaller as ∼324 Ω than 2D (∼1 kΩ). The ultrathin layered structure brings larger surface area to contact with electrolyte and less stacked layers give plenty of room for ion and electrons to transfer. The R_ct_ of Pt1/UT and Pt40/UT are also investigated. Unsurprisingly, with Pt element introduced, the arches decrease meaning that the R_ct_ obviously decreased to ∼31.7 Ω and ∼9.4 Ω, respectively. Noble metal largely increases the conductivity of UT. It still can be easily concluded with only 0.4 wt% Pt anchored on UT sheets, the conductivity is massively increased. The enhancement of conductivity combined with outstanding mass activity of Pt1/UT illustrate that UT can be an ideal material in all Ti_3_C_2_T_x_ based electrocatalyst.

To investigate the cycle stability of Pt1/UT, a chronopotentiometry test was performed. It shows obvious difference stability performance between Pt1/UT and Pt/C. The overpotential of Pt/C was at a lower value (60 mV) compared with Pt1/UT (71 mV). However, after 3 h of hydrogen evolution at constant current, the value turned to larger value of 88 mV while Pt1/UT stayed nearly unchanged (74 mV). This may be due to the instability of the structure of Pt/C during long HER cycle process. The overpotential retention rate is also shown in Fig. 5f for intuitive observation, from which the Pt1/UT retained ∼97% of overpotential while Pt/C reduced to below 60% after 3 h test. Furthermore, with C_dl_ calculated in Fig. S14a The value barely changes meaning the electrocatalyst still maintain its HER activity. The corresponding CV curves are shown in Fig. S14b and Fig. S14c. While for the C_dl_ of Pt/C in Fig. S15a, the collapse of Pt/C structure finally causes the reduction of ECSA. The corresponding CV curves of Pt/C are shown in Fig. S15b and Fig. S15c. Furthermore, the Pt1/UT MXene sheets after 3 h stability test had been investigated in Fig. S16.As is shown, the MXene sheets curl up together and TiO_2_ is formed on the surface due to the oxidization effect during the HER process. However, the majority of the MXene sheets stays in ultrathin form, which shows the stability of the structure with little restacking happened.

To further reveal the valence state change of Ti in Pt1/UT, an XPS spectra of Ti 2p is shown i n Fig. S17. The doublet peaks represent Ti^3+^ in Pt1/UT before the 3 h test shift to left side representing high binding energy. This means during the stability test, the ultrathin MXene sheets begin to being oxidized due to the oxygen dissolved in electrolyte evolved from counter electrode. This leads to the appearance of doublets representing TiO_2_ in Fig. S17.

## 4 Conclusions

In summary, a two-step etching in preparation of ultrathin MXene has been reported. The successfully prepared sample exhibits a rather large area of uniformly distributed and ultrathin MXene sheets with little restacking happens. This characteristic makes UT owns four times larger specific surface area than 2D according to BET result, which leads to much more electrochemical active sites. The C_dl_ result proves that UT can provide more active sites for HER than 2D with 65.5 mF cm^−2^ which is two times of C_dl_ of 2D. Furthermore, the prepared UT shows much better HER property (∼190 mV at 10 mA cm^−2^), smaller R_ct_ (∼324 Ω). Moreover, the Pt1/UT shows better HER performance (∼75 mV at 10 mA cm^−2^) and Tafel slope (∼61.7 mV·dec^−1^), which is close to the Tafel slope of 40 wt% commercial Pt/C (∼45.1 mV·dec^−1^). The Pt1/UT also performs well in cyclic stability test (∼75 mV at 10 mA cm^−2^ after 3 h chronopotentiometry test) while Pt/C maintain ∼88 mV overpotential at 10 mA cm^−2^ after 3 h test indicating better structural stability. The Pt40/UT even perform better (51 mV at 10 mA cm^−2^) than Pt/C in LSV test with 24 wt% Pt added. This study gives a way for synthesis of MXene with bigger specific surface area with more active sites. With little Pt anchored, the HER performance can be evidently improved. This work will improve the original MXene HER performance as substrate and exploit the potential in loading Pt particles.

## Author contribution statement

Zicong Yang: Conceived and designed the experiments; Performed the experiments; Analysed and interpreted the data; Wrote the paper. Yanhui Chen: Analysed and interpreted the data; Performed the experiments;Ge Chen: Wrote the paper; Analysed and interpreted the data. Jinshu Wang: contributed reagents, materials, analysis tools or data; wrote the paper. Hongyi Li: Conceived and designed the experiments; Wrote the paper; Contributed reagents, materials, analysis tools; Analysed and interpreted the data.

## Data availability statement

Data included in article/supp. Material/referenced in article.

## Declaration of competing interest

The authors declare that they have no known competing financial interests or personal relationships that could have appeared to influence the work reported in this paper.
