# Supplementary material for: Ultrathin Ti3C2Tx MXene sheets with high electrochemically active area anchored Pt boosting hydrogen evolution
Source: Heliyon. 2023 Aug 19;9(9):e19197. doi: 10.1016/j.heliyon.2023.e19197 (PMC10474418; doi:10.1016/j.heliyon.2023.e19197)
Supplement: Multimedia component 1 [file mmc1.docx]

**Ultrathin Ti_3_C_2_T_x_ MXene sheets with high electrochemically active area anchored Pt boosting hydrogen evolution**

**Supporting Information**


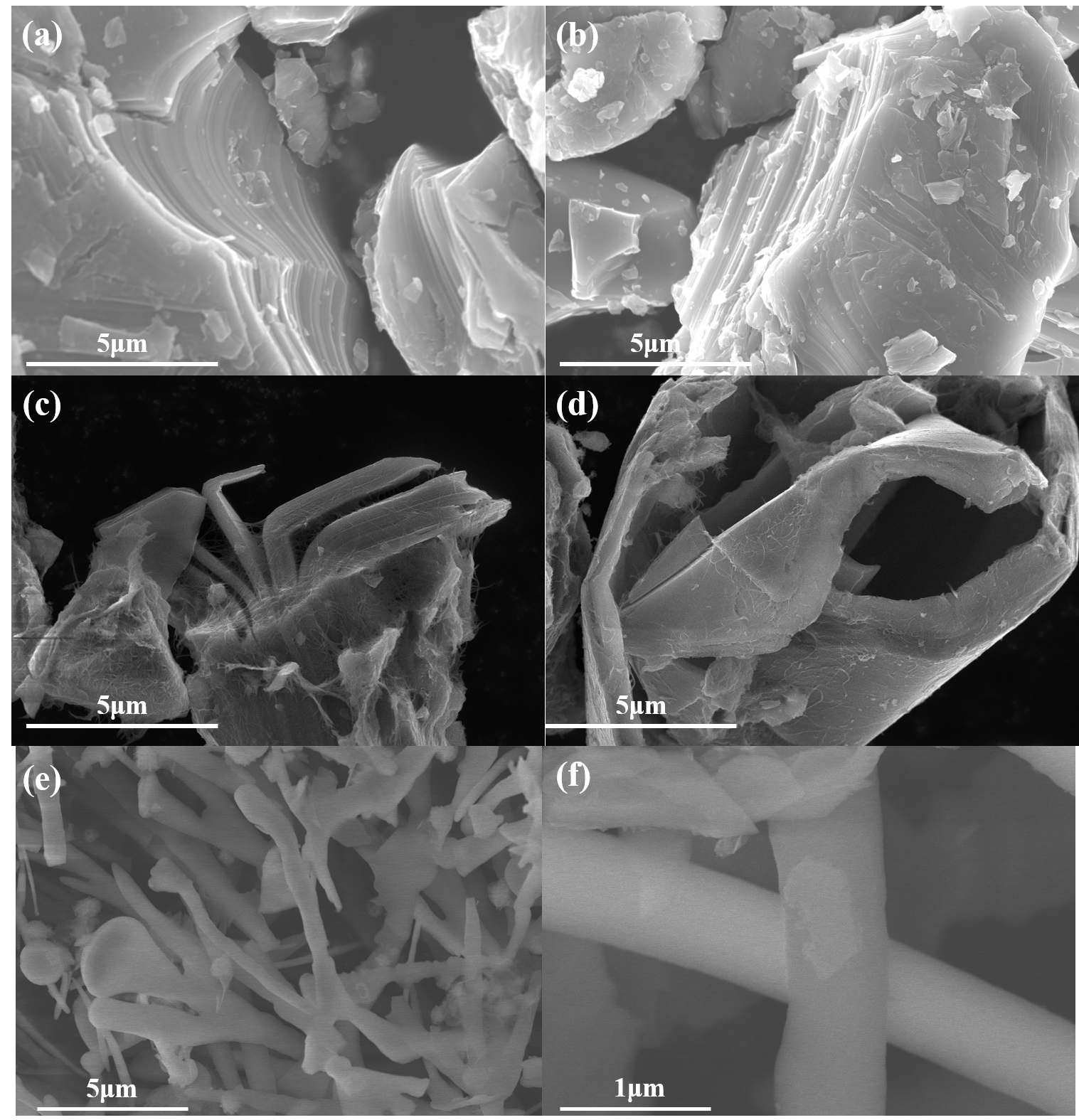


Fig. S1 a,b) SEM images of MAX precursor, c,d) 72h MAX precursor, e,f) MXene after 96h treatment with KOH


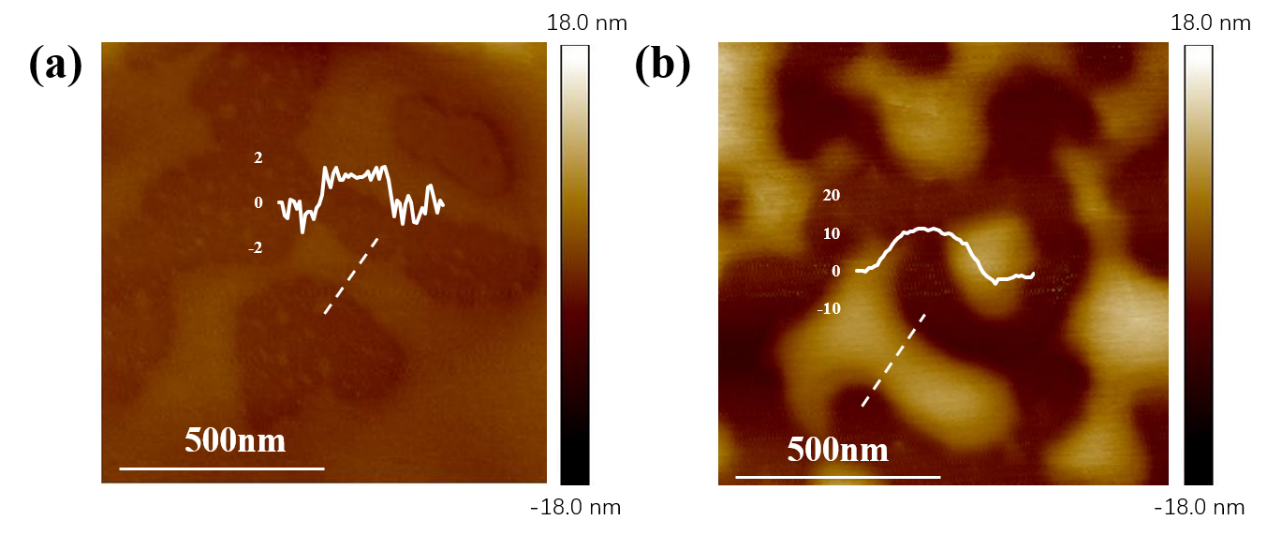
Fig. S2 AFM image and relevant thickness of (a) UT and (b) 2D.


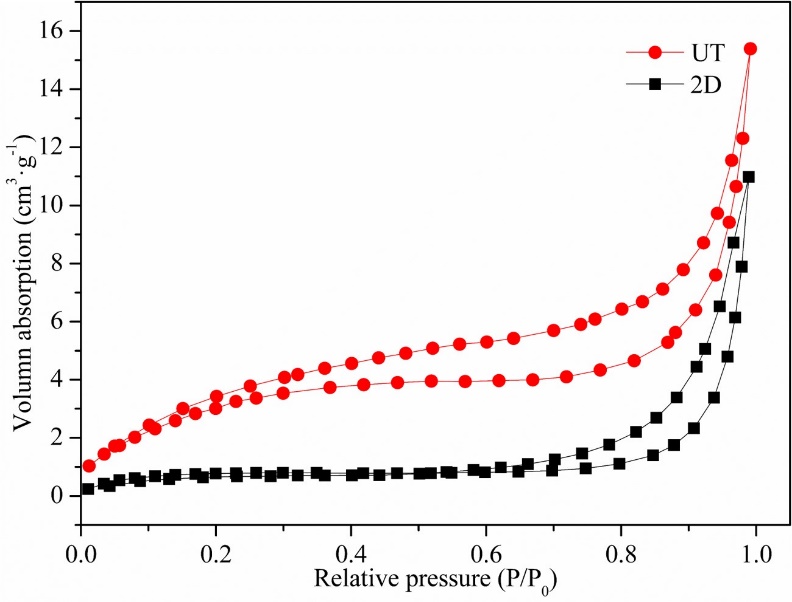


Fig. S3 The N_2_ absorption-desorption isotherm of 2D and UT.


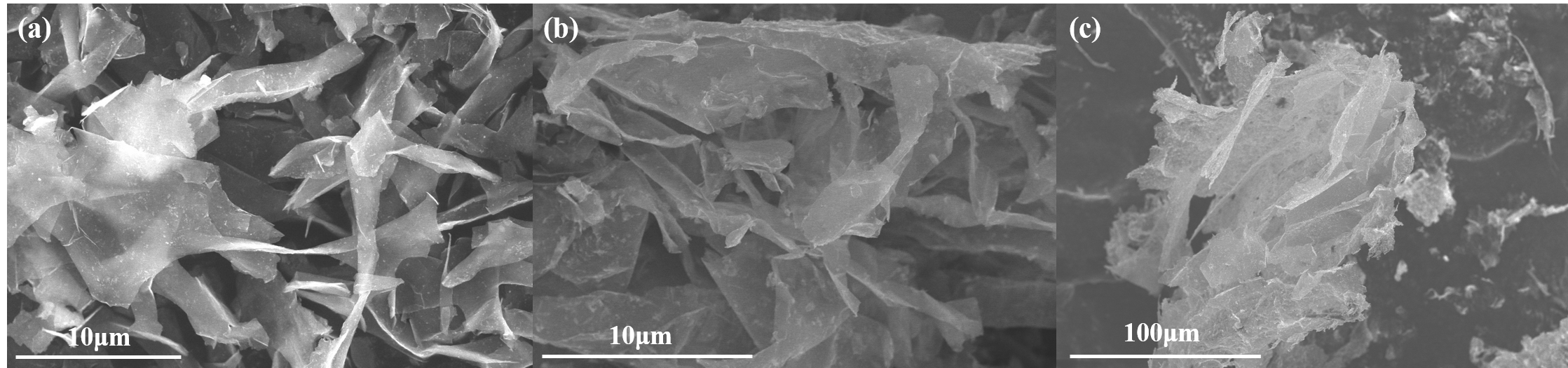


Fig. S4 SEM images of UT. Magnification: (a-b) 5k, (c) 500.


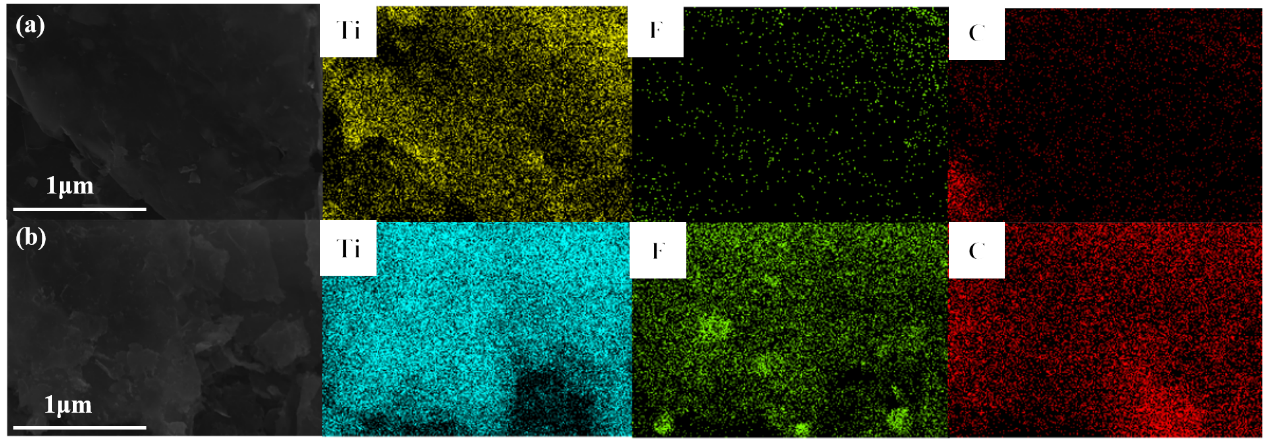


Fig. S5 Elemental mapping analysis for (a) UT, (b) 2D. Corresponding element: Ti, F, C.


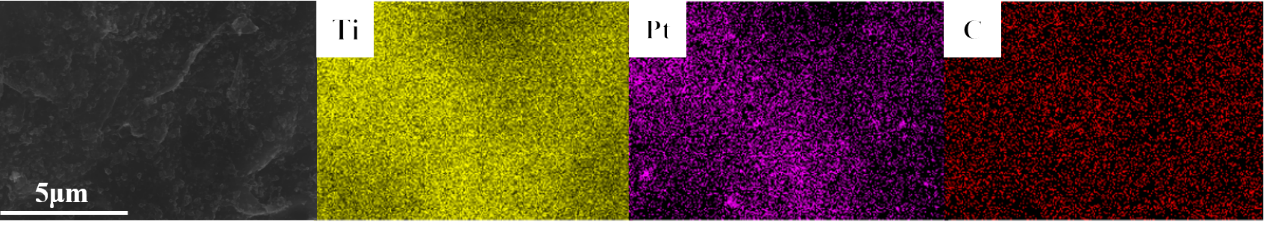


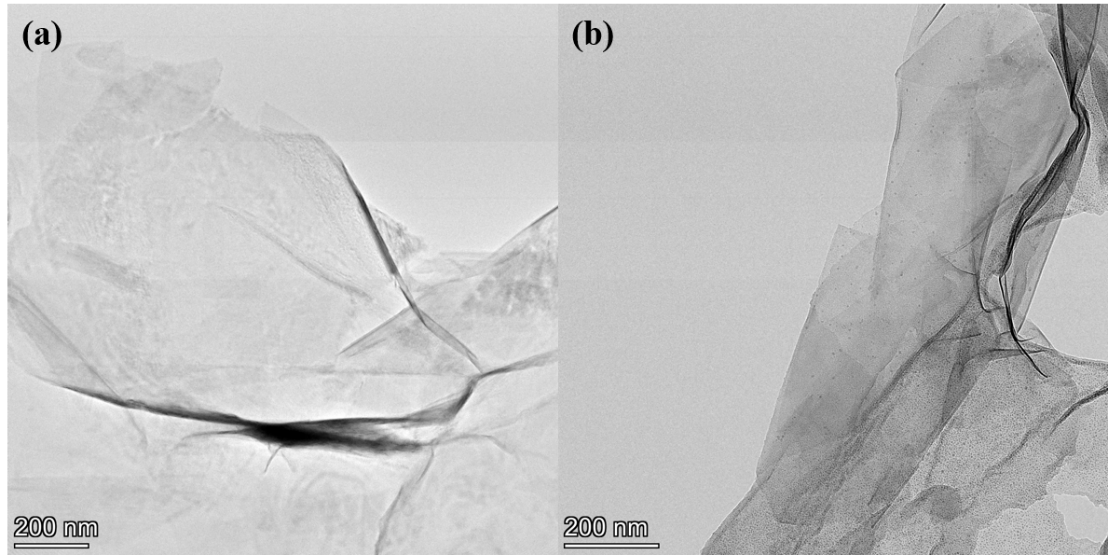
Fig. S6 Elemental mapping analysis of Pt10/UT with corresponding element Ti, Pt, C.

Fig. S7 TEM images of UT.


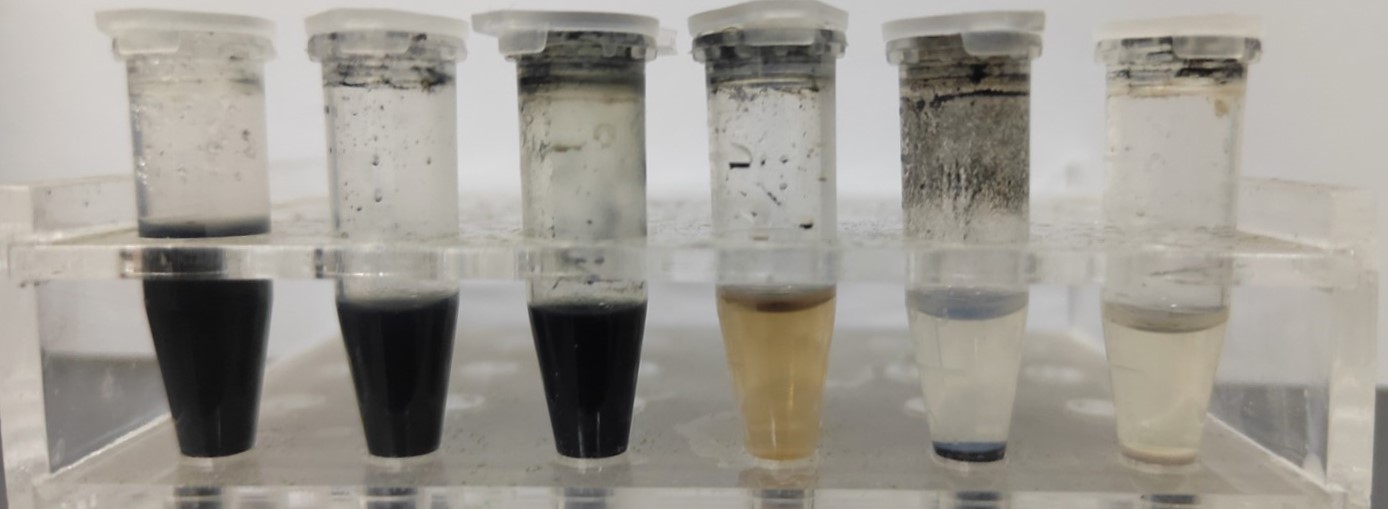


Fig. S8 photo of different samples after 48 hours stand. (left to right: 2D, UT, Pt1/UT, Pt1/2D, Pt10/UT, Pt40/UT)


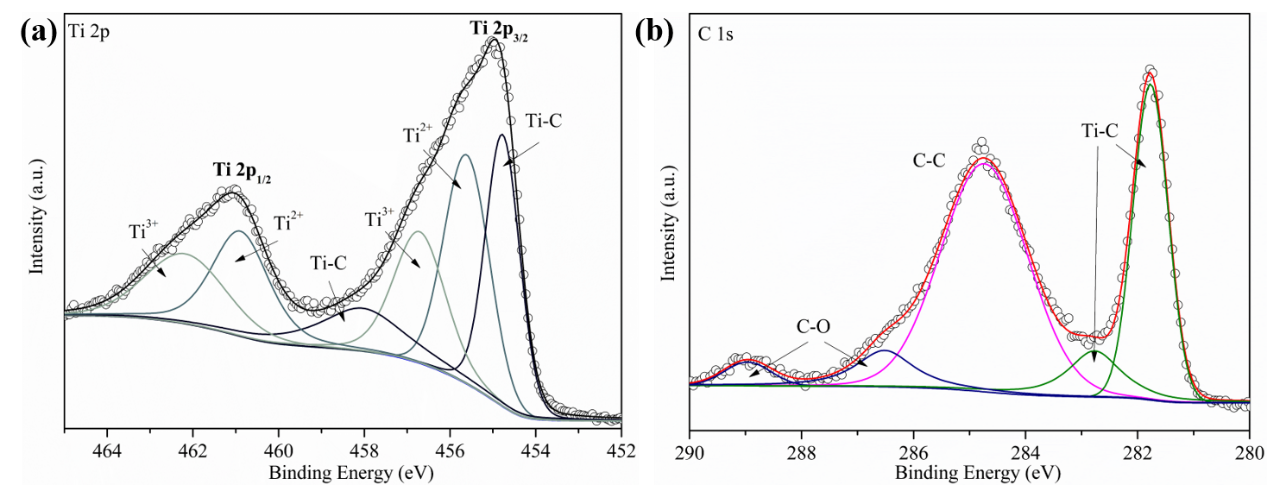


Fig. S9 XPS spectra of 2D (a)Ti 2p, (b) C1s.


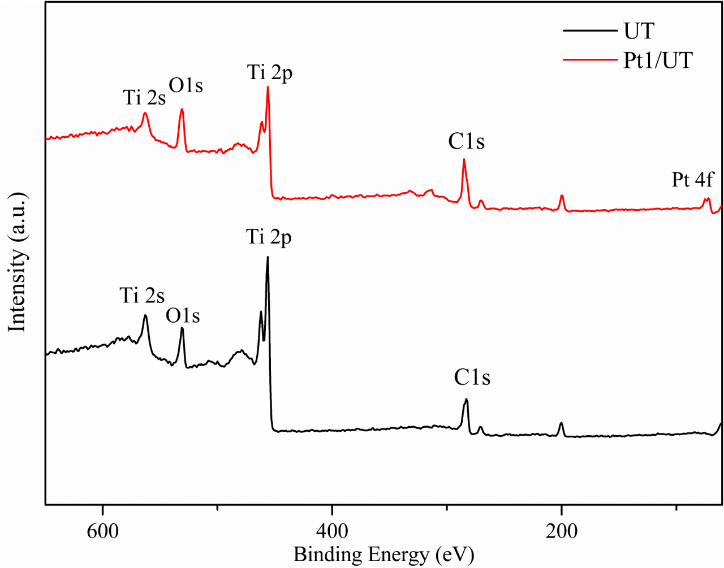


Fig. S10 Survey scan XPS spectra of UT and Pt1/UT.


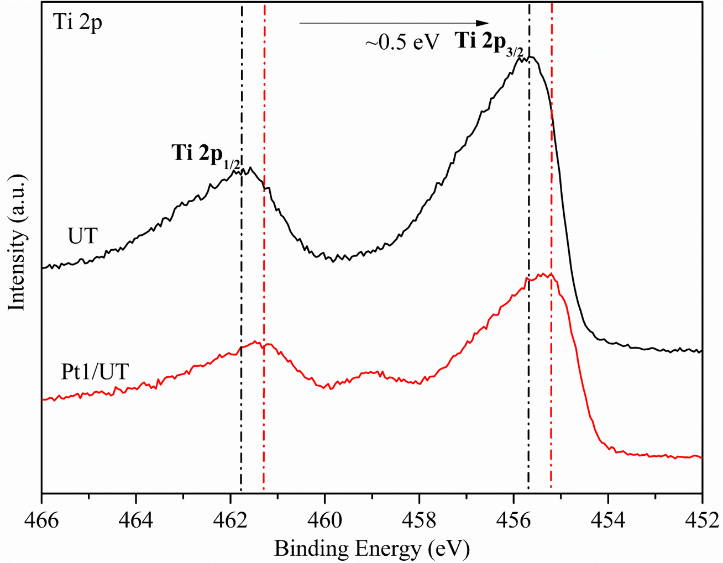


Fig. S11 XPS Ti 2p spectra of UT and Pt1/UT.





Fig. S12 Overpotential comparison of PT40/UT, PT10/UT, PT1/UT, UT and other previously reported noble metal-based or 2D material-based catalysts.[1-10]


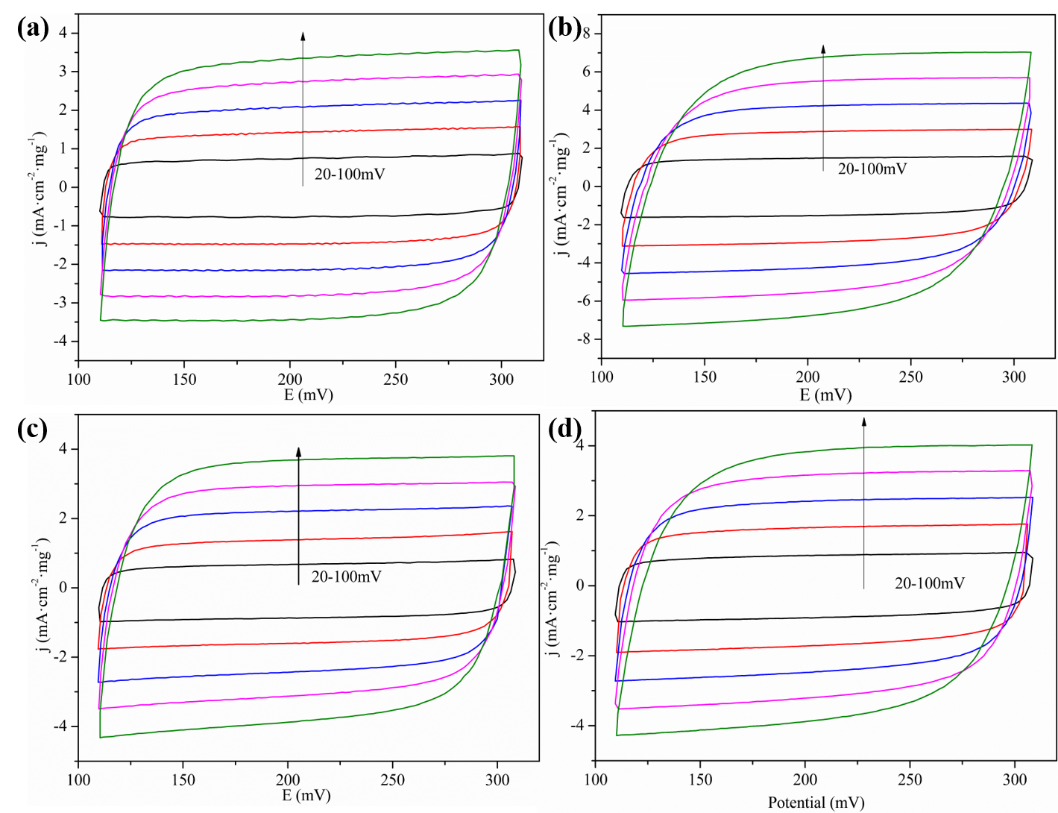
Fig. S13 CV curve at multiple scan rates for (a) 2D, (b) UT, (c) Pt/C, (d) Pt1/UT.


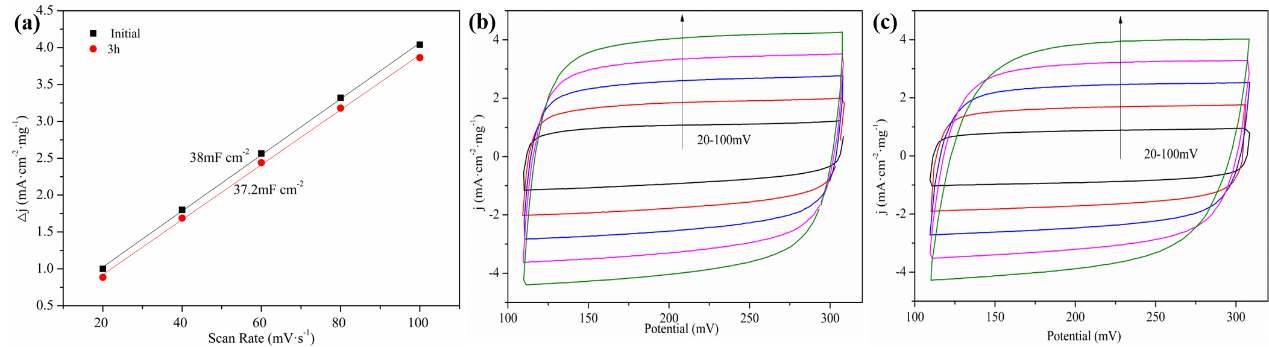


Fig. S14 (a) C_dl_ plots of Pt1/UT before and after 3h test. CV curves of Pt1/UT at multiple scan rate: (b) before 3h test, (c) after 3h test.


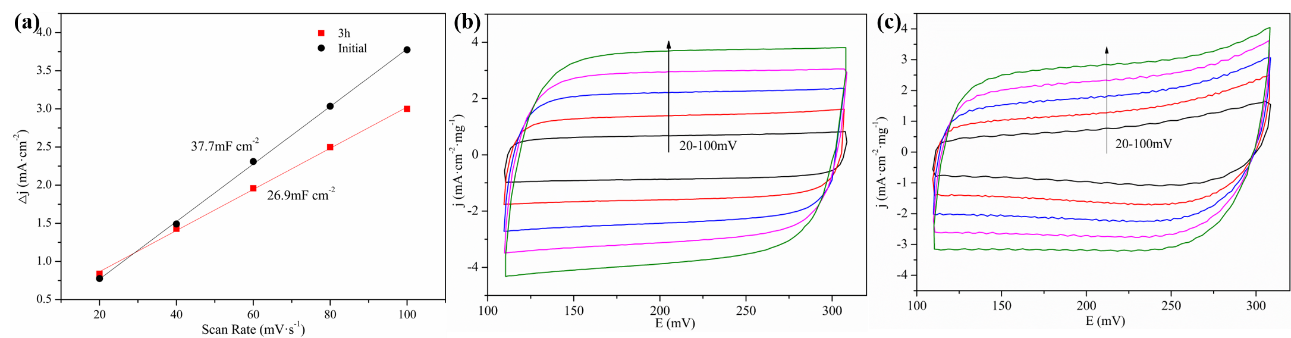


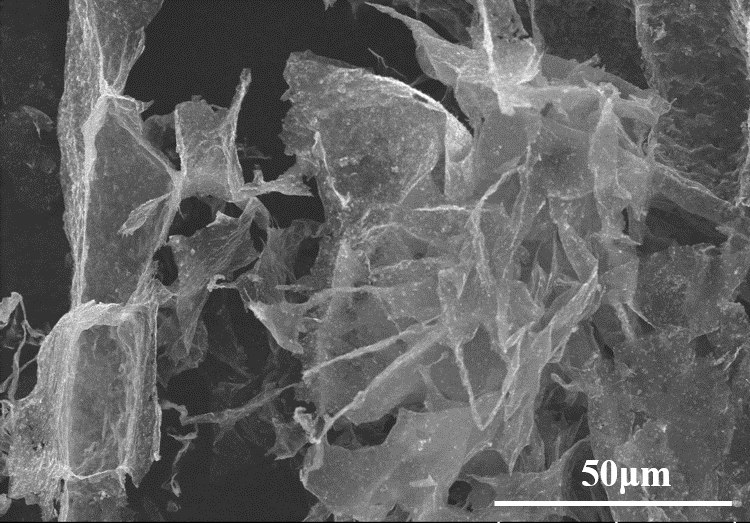
Fig. S15 (a) the HER polarization curves of Pt/C before and after 1000 cycles. (b) C_dl_ plots of Pt/C before and after 3h test. CV curves of multiple scan rates: (c) before 3h, (d) after 3h.

Fig. S16 Pt1/UT sheets after 3h chronopotentiometry test.





Fig. S17 Ti 2p XPS spectra of Pt1/UT before and after 3h test

**Reference**

[1] ZHANG X, SHAO B, SUN Z, GAO Z, QIN Y, et al. Platinum Nanoparticle-Deposited Ti3c2tx Mxene for Hydrogen Evolution Reaction[J]. Industrial & Engineering Chemistry Research, 2020,59(5): 1822-1828.

[2] RAMALINGAM V, VARADHAN P, FU H C, KIM H, ZHANG D, et al. Heteroatom-Mediated Interactions between Ruthenium Single Atoms and an Mxene Support for Efficient Hydrogen Evolution[J]. Adv Mater, 2019,31(48): e1903841.

[3] LUO Z, OUYANG Y, ZHANG H, XIAO M, GE J, et al. Chemically Activating Mos2 Via Spontaneous Atomic Palladium Interfacial Doping Towards Efficient Hydrogen Evolution[J]. Nat Commun, 2018,9(1): 2120.

[4] DENG J, LI H, XIAO J, TU Y, DENG D, et al. Triggering the Electrocatalytic Hydrogen Evolution Activity of the Inert Two-Dimensional Mos2 Surface Via Single-Atom Metal Doping[J]. Energy & Environmental Science, 2015,8(5): 1594-1601.

[5] XUAN N, CHEN J, SHI J, YUE Y, ZHUANG P, et al. Single-Atom Electroplating on Two Dimensional Materials[J]. Chemistry of Materials, 2018,31(2): 429-435.

[6] YIN X P, WANG H J, TANG S F, LU X L, SHU M, et al. Engineering the Coordination Environment of Single-Atom Platinum Anchored on Graphdiyne for Optimizing Electrocatalytic Hydrogen Evolution[J]. Angew Chem Int Ed Engl, 2018,57(30): 9382-9386.

[7] Q.M., M. LI, ZI. W, LIU X, ZHU X, et al. Pt Monolayer Coating on Complex Network Substrate with High Catalytic Activity for the Hydrogen Evolution Reaction[J]. 2015.

[8] Z. Chen, J. Lu, Y. Ai, Y. Ji, T. Adschiri, L. Wan, Ruthenium/Graphene-like Layered Carbon Composite as an Efficient Hydrogen Evolution Reaction Electrocatalyst, ACS Appl Mater Interfaces, 8 (2016) 35132-35137.

[9] J. Zhang, E. Wang, S. Cui, S. Yang, X. Zou, Y. Gong, Single-Atom Pt Anchored on Oxygen Vacancy of Monolayer Ti3C2Tx for Superior Hydrogen Evolution, Nano Lett, 22 (2022) 1398-1405.

[10] Y. Wu, W. Wei, R. Yu, L. Xia, X. Hong, J. Zhu, J. Li, L. Lv, W. Chen, Y. Zhao, L. Zhou, L. Mai, Anchoring Sub‐Nanometer Pt Clusters on Crumpled Paper‐Like MXene Enables High Hydrogen Evolution Mass Activity, Advanced Functional Materials, 32 (2022).
